# Supplementary material for: Intravenous administration of BCG in mice promotes natural killer and T cell-mediated antitumor immunity in the lung
Source: Nat Commun. 2023 Oct 4;14:6090. doi: 10.1038/s41467-023-41768-8 (PMC10551006; doi:10.1038/s41467-023-41768-8)
Supplement: Supplementary file 1 — Supplementary Information [file 41467_2023_41768_MOESM1_ESM.pdf]

SUPPLEMENTARY INFORMATION

Intravenous administration of BCG in mice promotes natural killer and T cell-mediated antitumor immunity in the lung

Eduardo Moreo, Aitor Jarit-Cabanillas, Iñaki Robles-Vera, Santiago Uranga, Claudia Guerrero, Ana Belén Gómez, Pablo Mata-Martínez, Luna Minute, Miguel Araujo-Voces, María José Felgueres, Gloria Esteso, Iratxe Uranga-Murillo, Maykel Arias, Julián Pardo, Carlos Martín, Mar Vales-Gómez, Carlos del Fresno, David Sancho and Nacho Aguiló

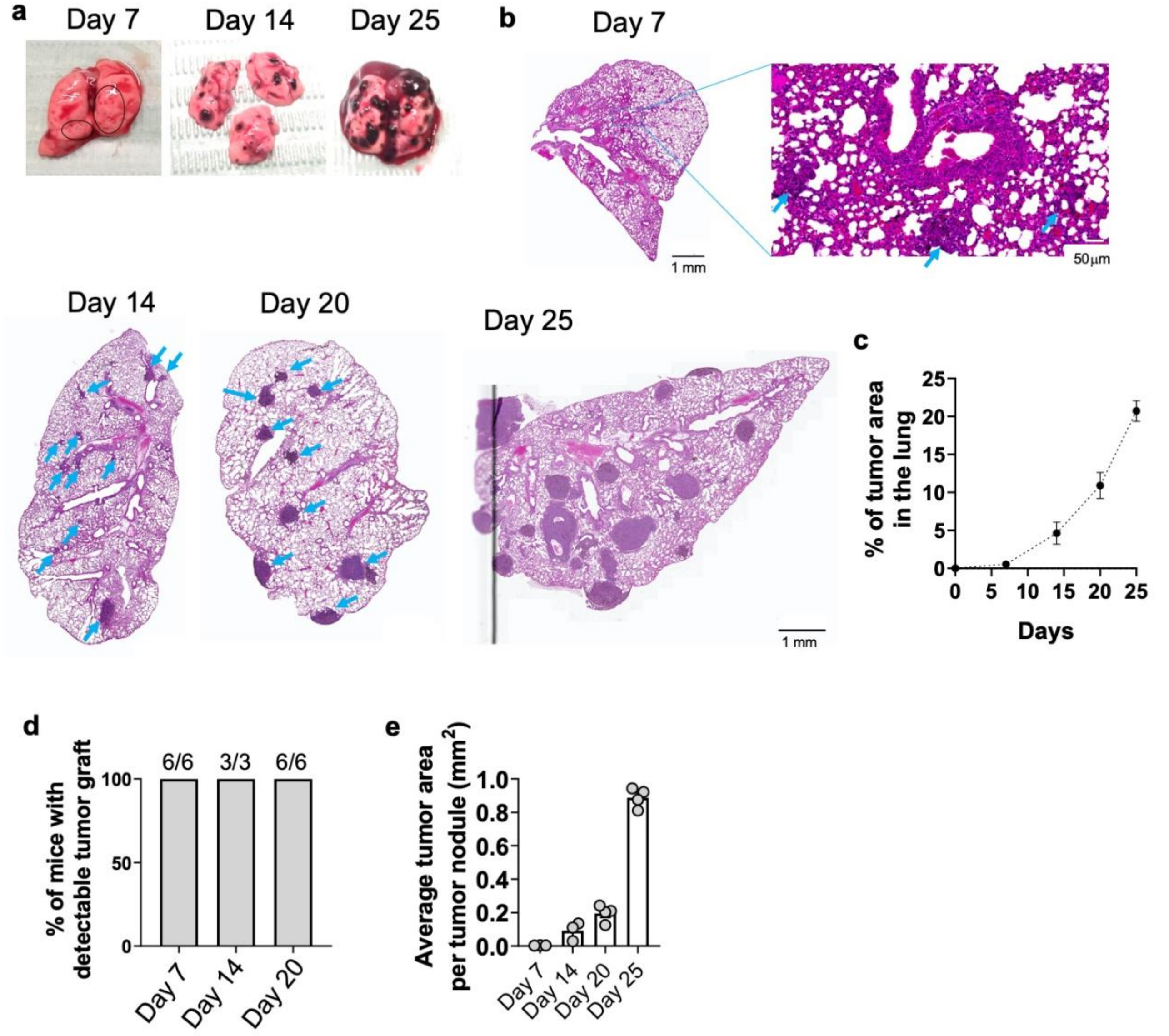

Supplementary Figure 1: Progression of B16-F10 lung metastases following IV inoculation. a, b Representative images of lungs are shown **(a)** and lung tissue sections stained with H&E **(b)** from B16-F10 tumor-bearing mice at different timepoints following inoculation. In (**a**), black circles surround areas with visible small tumor nodules. In **(b)**, blue arrows point three tumor nodules. **c,** Quantification of the % of lung covered by tumor at different timepoints post B16-F10 IV inoculation. **d,** Quantification of the % of mice with detectable tumor nodules in the lung. **e,** Quantification of the average tumor nodule area at different timepoints post inoculation. *n* = 3 mice (Days 7, 14) and *n* = 4 mice (Days 20, 25). IV: intravenous.

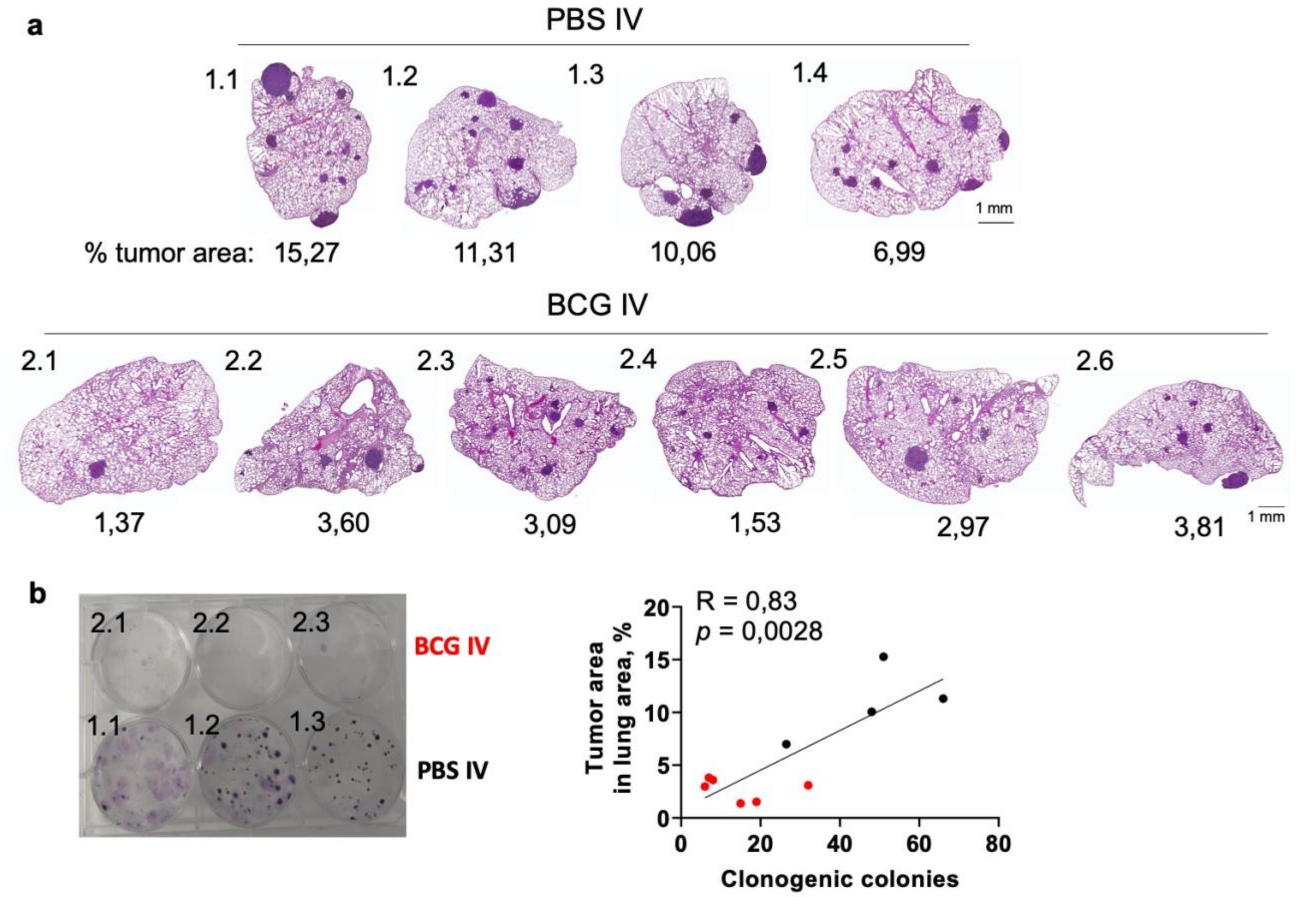

Supplementary Figure 2: Antitumoral effect of IV BCG in the B16-F10 model. a, Representative lung tissue sections stained with H&E from B16-F10 tumor-bearing mice (from **Figure 1d**). Below each image, the % of lung area covered by tumor is shown. **b,** Representative image from the clonogenic assay with lung single cell suspensions shown in **Figure 1e,** and correlation between the % of lung covered by tumor and the number of clonogenic colonies obtained by this method in each mouse**.** R square and *P* values were calculated by fitting the data with a linear regression model. PBS: phosphate-buffered saline. IV: intravenous, PBS: phosphate-buffered saline.

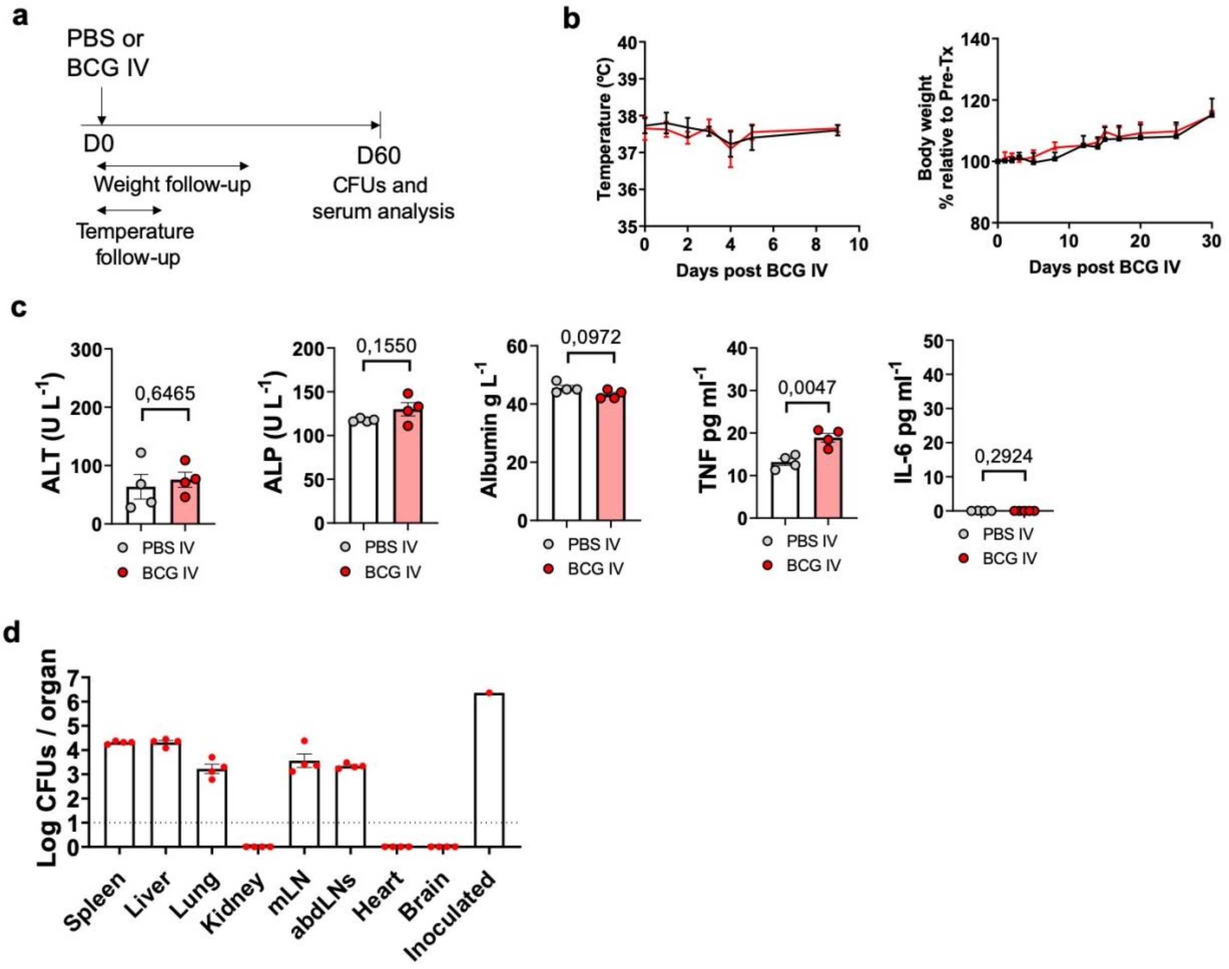

Supplementary Figure 3: Safety of IV BCG on tumor-free mice. a, Schematic diagram showing treatment strategy. **b,** Follow-up of mice body temperature (left) or weight (right) in the days following IV BCG inoculation (*n* = 4 mice/group, from one experiment). **c,** Serum levels of ALT (alanine transaminase), ALP (alkaline phosphatase), albumin, IL-6 and TNF at the day 60 endpoint (n = 4 mice/group, from one experiment). **d,** BCG CFUs cultured from the indicated organs at the day 60 endpoint, *n* = 4 mice, from one experiment. *P* values were calculated using two-tailed unpaired Student's t-test at a 95 % CI (**c**). Data is depicted as mean +/- SEM. mLN: mediastinal lymph node, TNF: tumor necrosis factor, abdLNs: abdominal lymph nodes, CFUs: colony forming units, ALT: alanine aminotransferase, ALP: alkaline phosphatase, IV: intravenous, PBS: phosphate-buffered saline.

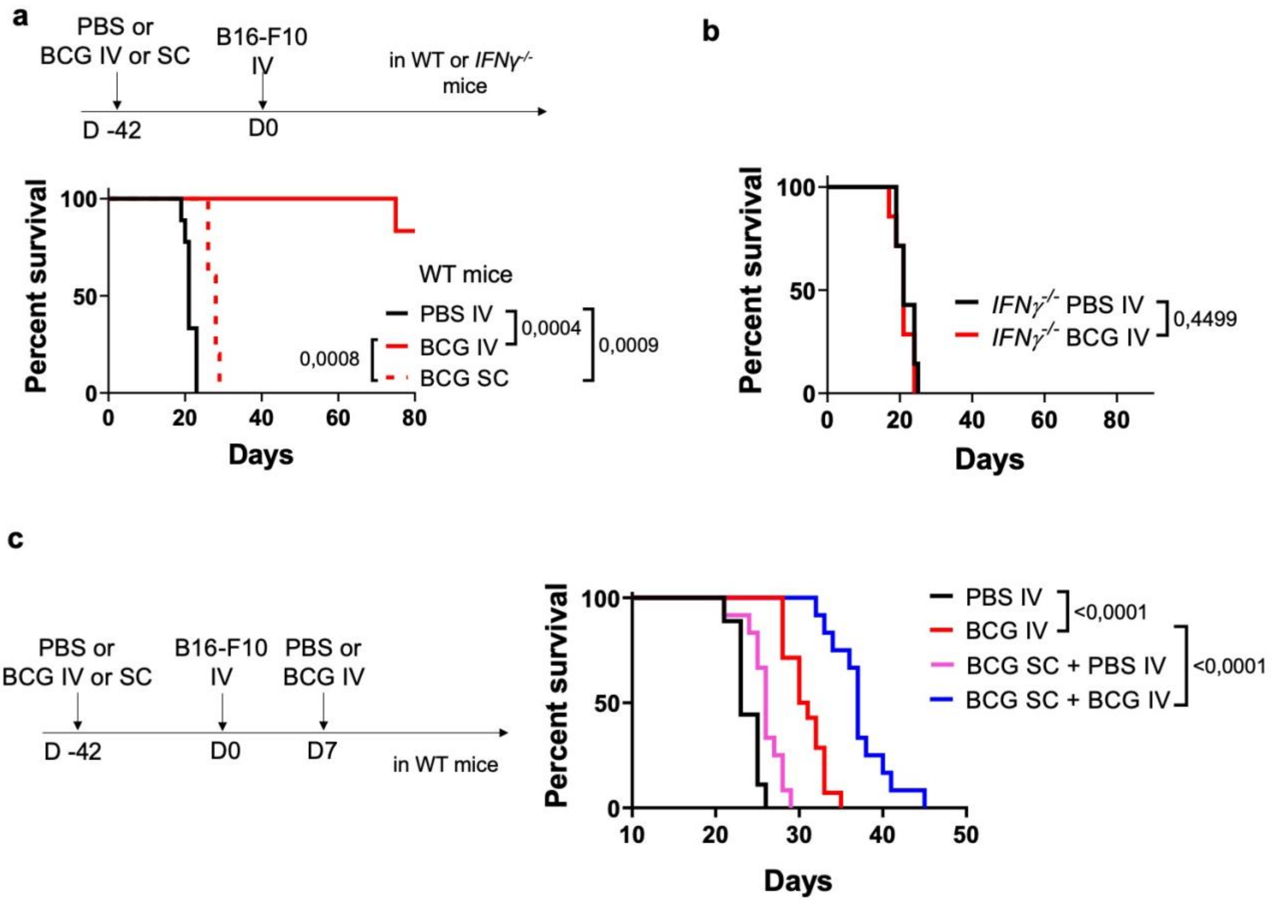

Supplementary Figure 4: Effect of previous BCG vaccination on the growth of B16-F10 lung metastases and on subsequent IV BCG treatment. a, Survival curve of mice vaccinated with BCG either subcutaneously (SC) or intravenously (IV) 42 days before B16-F10 IV tumor challenge (*n* = 9 mice for PBS IV, *n* = 6 mice for BCG SC and BCG IV, from one experiment). **b,** Survival curve of IFN-γ deficient (*IFNγ*$^{-/-}$) mice vaccinated with IV BCG 42 days before B16-F10 IV tumor challenge (*n* = 7 mice/group, from one experiment). **c,** Survival curve of mice vaccinated with either SC PBS or BCG 42 days before B16-F10 IV tumor challenge, and then treated with either IV PBS or BCG at day 7 post tumor cell implantation (*n* = 9 mice for PBS IV, *n* = 14 mice for BCG IV and *n* = 12 mice/group for BCG SC + BCG IV and BCG SC + PBS IV, pooled from two independent experiments). *P* values were calculated using Log-rank (Mantel-Cox) test (**a-c).** IV: intravenous, PBS: phosphate-buffered saline.

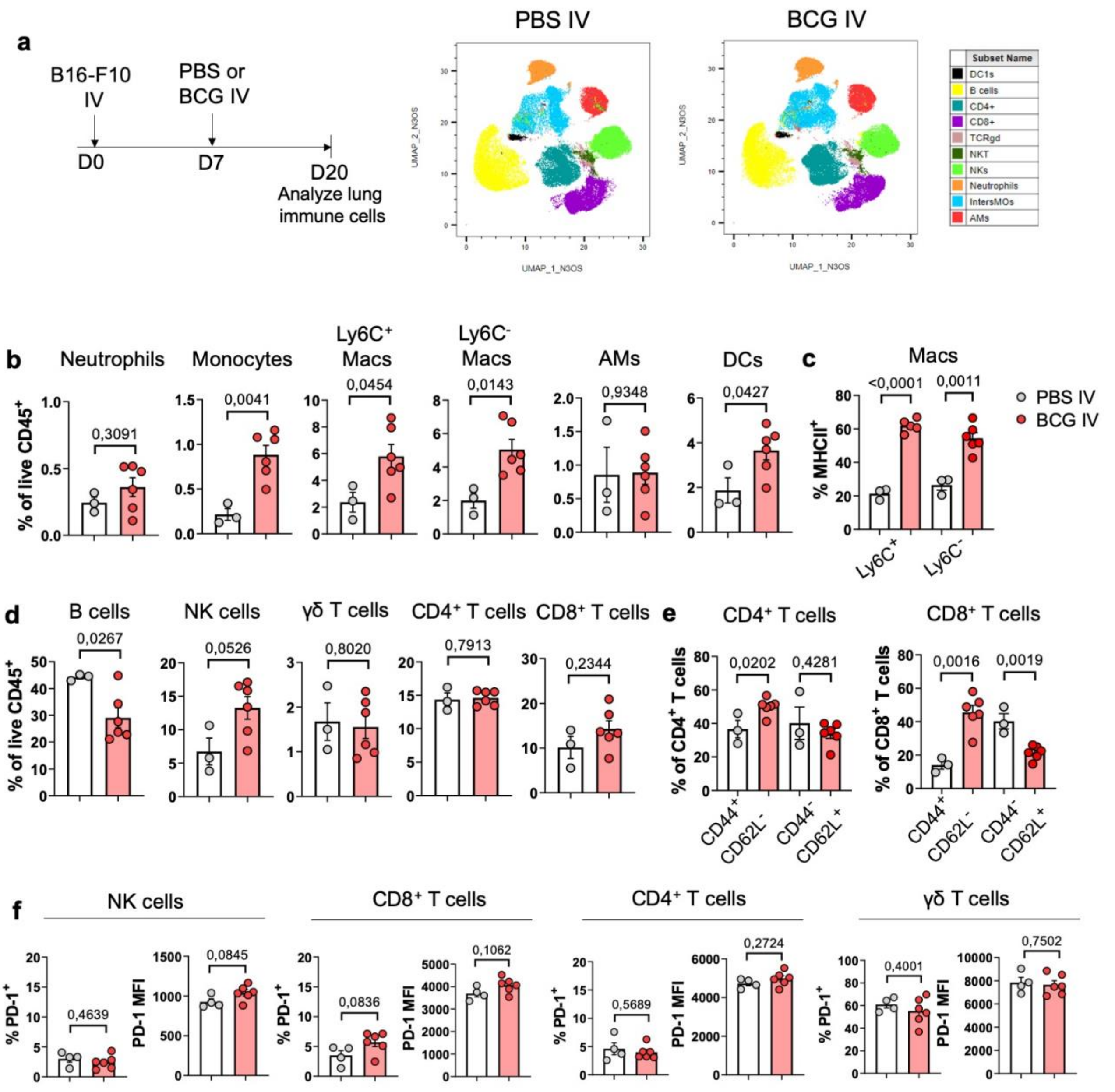

Supplementary Figure 5: Global analysis of B16-F10 lung metastases immune infiltrate. a, UMAP plots of single cells from the lungs of mice bearing B16-F10 lung metastases, treated with either IV PBS (n = 3 mice) or BCG (n = 6 mice) at day 7 and analyzed by spectral flow cytometry at day 20. **b,** Quantification of neutrophil, monocyte, macrophage and dendritic cell (DC) frequencies as % of live CD45^{+} cells. **c,** MHC-II expression by lung Ly6C^{+} AND Ly6C^{-} macrophages. **d,** Quantification of B cell, NK cells, γδ T cell, CD4^{+} T cell and CD8^{+} T cell frequencies as % of live CD45^{+} cells. **e,** CD44 and CD62L expression by CD4^{+} and CD8^{+} T cells. **f,** PD-1 expression by NK cells, CD8^{+} T cells, CD4^{+} T cells and γδ T cells. *P* values were calculated using two-tailed unpaired Student's t-test at a 95 % CI (**b-f**). Data depicted as mean +/- SEM. PBS: phosphate-buffered saline, IV: intravenous. PD-1: programmed cell death 1.

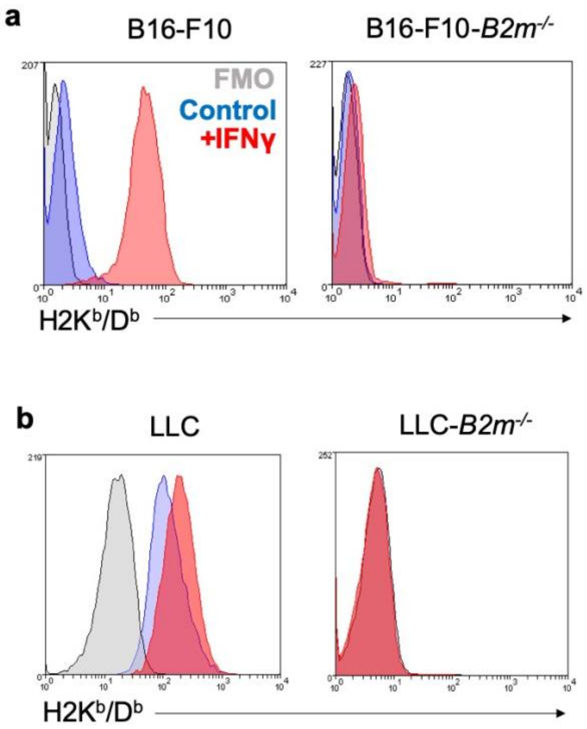

Supplementary Figure 6: Validation of B16-F10-*B2m*$^{-/-}$ and LLC- *B2m*$^{-/-}$ tumor cells. a, Parental B16-F10 and B16-F10-*B2m*$^{-/-}$ or parental LLC and LLC-*B2m*$^{-/-}$ **(b)** were incubated for 24 h in the presence or not of 10 ng ml^{-1} mouse IFN-γ, stained for H2K^b/D^b expression and analysed by flow cytometry. Representative histograms of two independent experiments are shown. B2m: beta2 microglobulin.

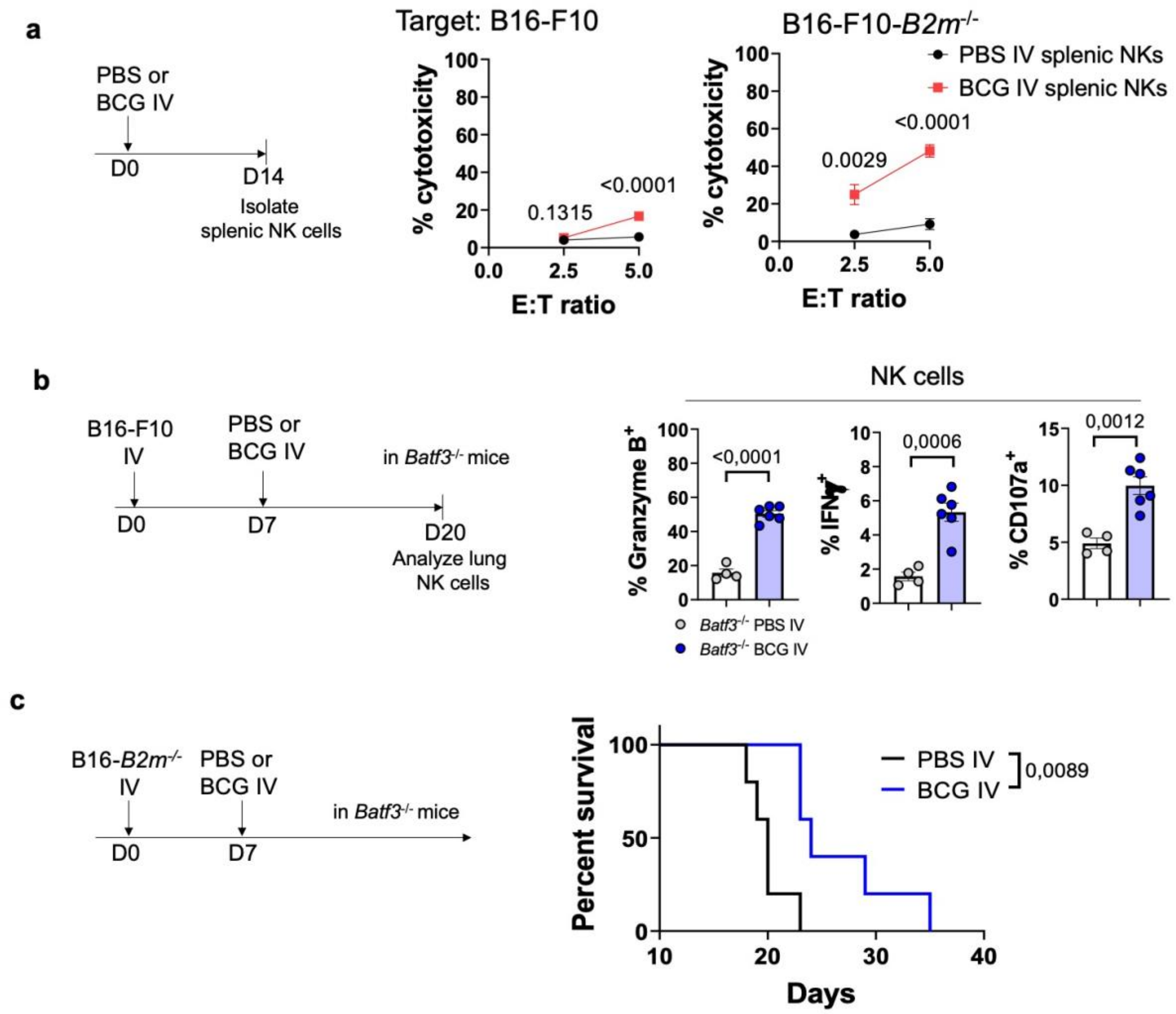

Supplementary Figure 7: Activation of NK cells by IV BCG. **a,** Cytotoxicity of spleen NK cells from control or IV BCG-treated mice against parental B16-F10 cells (left) or B16-F10-*B2m*$^{-/-}$ (right) at two different E:T ratios. The percentage of dying (Annexin V^{+} 7-AAD^{-}) and dead (Annexin V^{+} 7-AAD^{+}) target tumor cells after 20 h of coincubation was analysed by flow cytometry. Data pooled from three independent experiments, with NK cells pooled from n = 2 mice per group in each experiment and each condition run in duplicate. **b,** Expression of Granzyme B, IFN-γ and CD107a in NK cells from the lungs of *Batf3*$^{-/-}$ mice bearing B16-F10 lung metastases at day 20, n = 4 mice for PBS IV and n = 6 mice for BCG IV, from one experiment. **c,** Survival of *Batf3*$^{-/-}$ mice bearing B16-F10-*B2m*$^{-/-}$ lung metastases and treated with IV PBS or BCG (n = 6 mice/group, from one experiment). *P* values were calculated using two-tailed unpaired Student's t-test at a 95 % CI (**a, b**), log-rank (Mantel-Cox) test (**c**)**.** Data depicted as mean +/- SEM. PBS: phosphate-buffered saline, B2m: beta2 microglobulin, IV: intravenous.

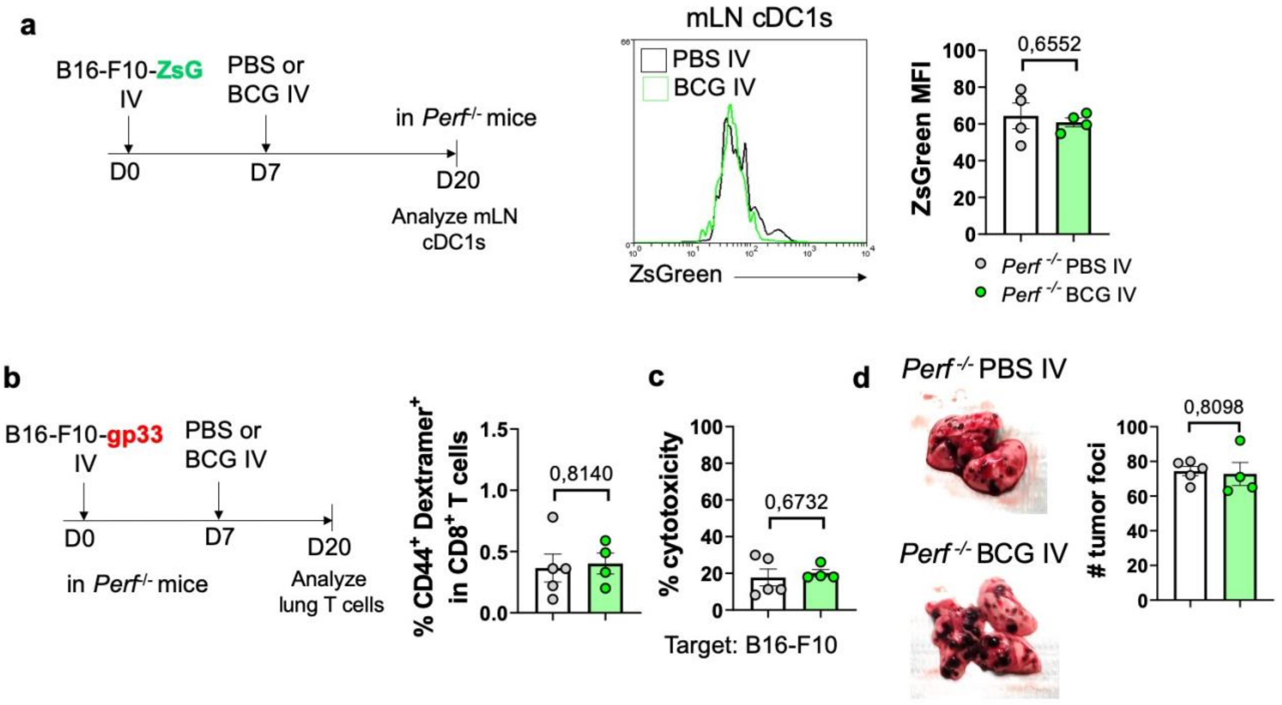

Supplementary Figure 8: Perforin mediated killing of tumor cells is necessary to unleash the BCG-induced tumor-specific adaptive immune response. **a,** ZsGreen expression by cDC1s from the lung-draining mediastinal lymph node (mLN) of *Perf*$^{-/-}$ mice bearing ZsGreen-expressing B16-F10 lung tumors at day 20 (*n* = 4 mice per group, from one experiment). **b,** Quantification of CD44^{+} gp33-specific CD8^{+} T cells in the lungs of *Perf*$^{-/-}$ mice bearing gp33-expressing B16-F10 lung tumors at day 20 (*n* = 5 mice for PBS IV and n = 4 mice for BCG IV, from one experiment). **c,** Cytotoxicity of splenocytes isolated from the indicated groups of mice bearing B16-F10.gp33 lung tumors against target B16-F10.ZsGreenLuc and LLC-ZsGreenLuc cells incubated at a 100:1 effector to target ratio. Percentage cytotoxicity was calculated in reference to the luminescence emitted by cells cultured without splenocytes (*n* = 5 mice for PBS IV and n = 4 mice for BCG IV, from one experiment). **d,** Representative images and quantification of the number of B16-F10-gp33 lung surface metastases at day 20 in *Perf*$^{-/-}$ mice (*n* = 5 mice for PBS IV and n = 4 mice for BCG IV, from one experiment). *P* values were calculated using two-tailed unpaired Student's t-test at a 95 % CI (**a-d**). Data depicted as mean +/- SEM. Perf: perforin, ZsG: ZsGreen, mLN: mediastinal lymph node, cDC1: type 1 conventional dendritic cell, PBS: phosphate-buffered saline, IV: intravenous.

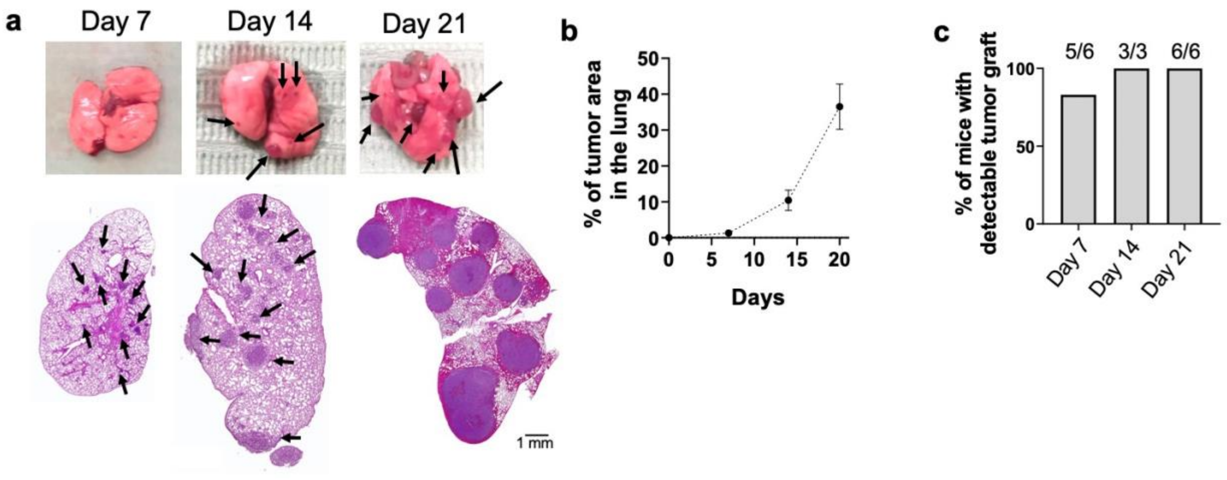

Supplementary Figure 9: Progression of orthotopic LLC lung tumors. a, Representative images of lungs are shown **(a)** and lung tissue sections stained with H&E **(b)** from orthotopic LLC tumor-bearing mice at different timepoints following inoculation. In (**a**), black arrows mark visible tumor nodules. **b,** Quantification of the percentage of lung area covered by tumor at different timepoints post LLC IV inoculation. **c,** Percentage of mice with detectable LLC tumor nodules in the lung. *n* = 3 mice for day 7, *n* = 4 mice for day 14 and *n* = 6 mice for day 20. IV: intravenous.

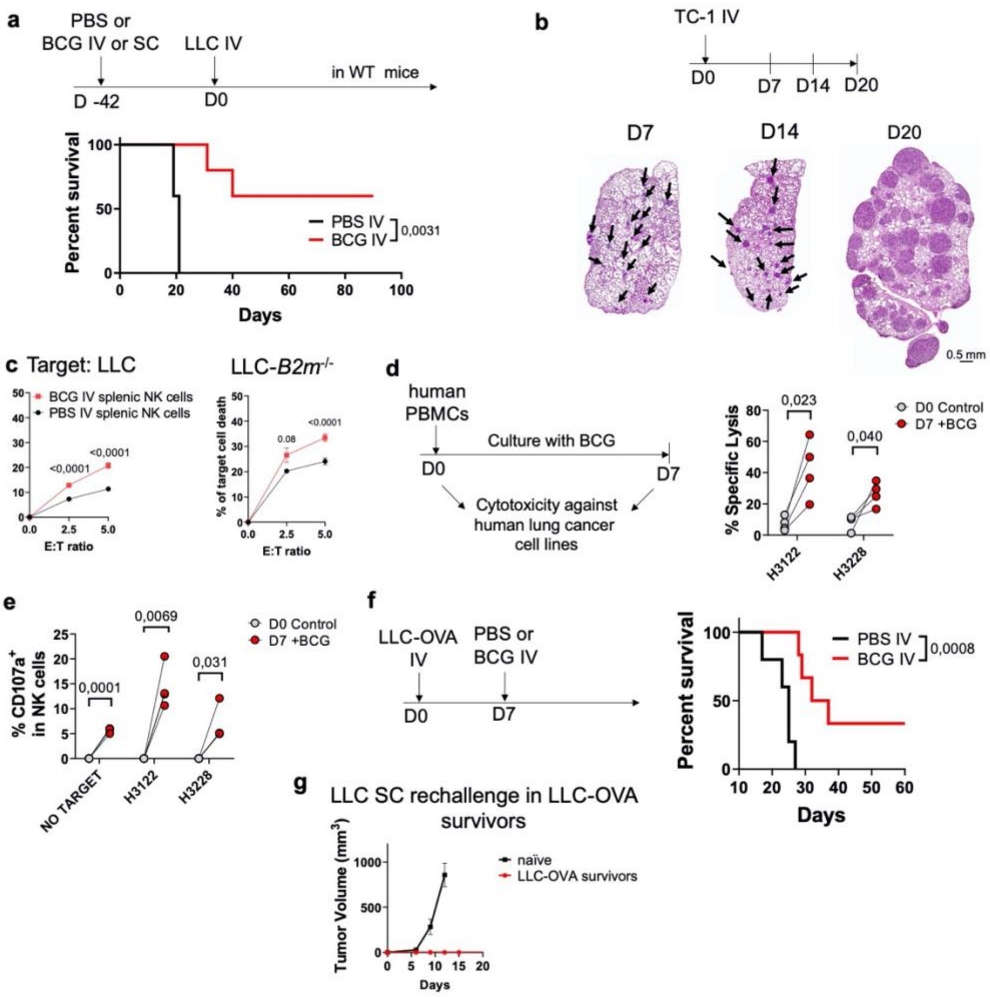

Supplementary Figure 10: Antitumoral effect of IV BCG in the orthotopic LLC model. a, Survival curves of mice vaccinated with IV PBS or BCG 42 prior to LLC orthotopic tumor implantation (*n* = 5 mice/group, from one experiment). **b,** Images of lung tissue sections stained with H&E from orthotopic TC-1 tumor-bearing mice at different timepoints following inoculation are shown (representative of *n* = 3 mice per timepoint). **c,** Cytotoxicity of spleen NK cells from control or IV BCG-treated mice against parental LLC cells (left) or LLC-*B2m*$^{-/-}$ (right) at two different E:T ratios. The percentage of dying (Annexin V^{+} 7-AAD^{-}) and dead (Annexin V^{+} 7-AAD^{+}) target tumor cells after 20 h of incubation was analysed by flow cytometry. Data pooled from two independent experiments, with NK cells pooled from *n* = 2 mice per group per experiment and each condition run in triplicates. **d,** Cytotoxicity exerted by unstimulated or BCG-stimulated human PBMCs against H3122 and H3228 human lung cancer cell lines. **e,** Flow cytometry analysis of CD107a expression by NK cells in the cytotoxicity assay shown in **(d)**. **f,** Survival of mice bearing orthotopic LLC-OVA lung tumors and treated with IV PBS or BCG at day 7 (*n* = 6 mice/group, from one experiment). **g,** Mice which survived orthotopic LLC-OVA tumors were subcutaneously rechallenged (at day 70 post primary tumor inoculation) with LLC cells, and naïve mice were used as controls. *P* values were calculated using two-tailed unpaired Student's t-test at a 95 % CI (**c**), two-tailed paired Student's t-test at a 95 % CI (**d, e**), log-rank (Mantel-Cox) test (**a, f).** Data depicted as mean +/- SEM. PBMCs: peripheral blood mononuclear cells, PBS: phosphate-buffered saline, OVA: ovalbumin, SC: subcutaneous, WT: wild-type, B2m: beta2 microglobulin, IV: intravenous.

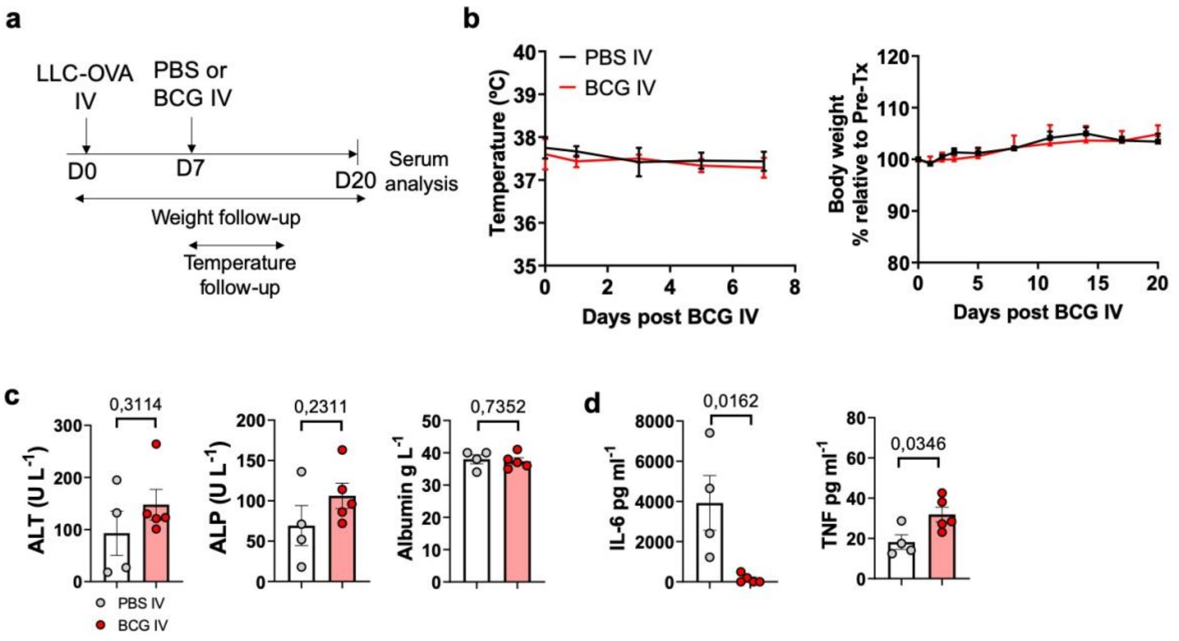

Supplementary Figure 11: Safety of IV BCG in LLC-tumor bearing mice. a, Schematic diagram showing treatment strategy. **b,** Follow-up of mice body temperature (left) or weight (right) in the days following IV BCG inoculation (*n* = 4 mice for PBS IV and *n* = 5 mice for BCG IV, from one experiment). **c,d** Serum levels of ALT (alanine transaminase), ALP (alkaline phosphatase), albumin, IL-6 and TNF at the day 20 endpoint (*n* = 4 mice for PBS IV and *n* = 5 mice for BCG IV, from one experiment). *P* values were calculated using two-tailed unpaired Student's t-test at a 95 % CI (**c,d)**. Data is depicted as mean +/- SEM. TNF: tumor necrosis factor, PBS: phosphate-buffered saline, ALT: alanine aminotransferase, ALP: alkaline phosphatase, IV: intravenous.

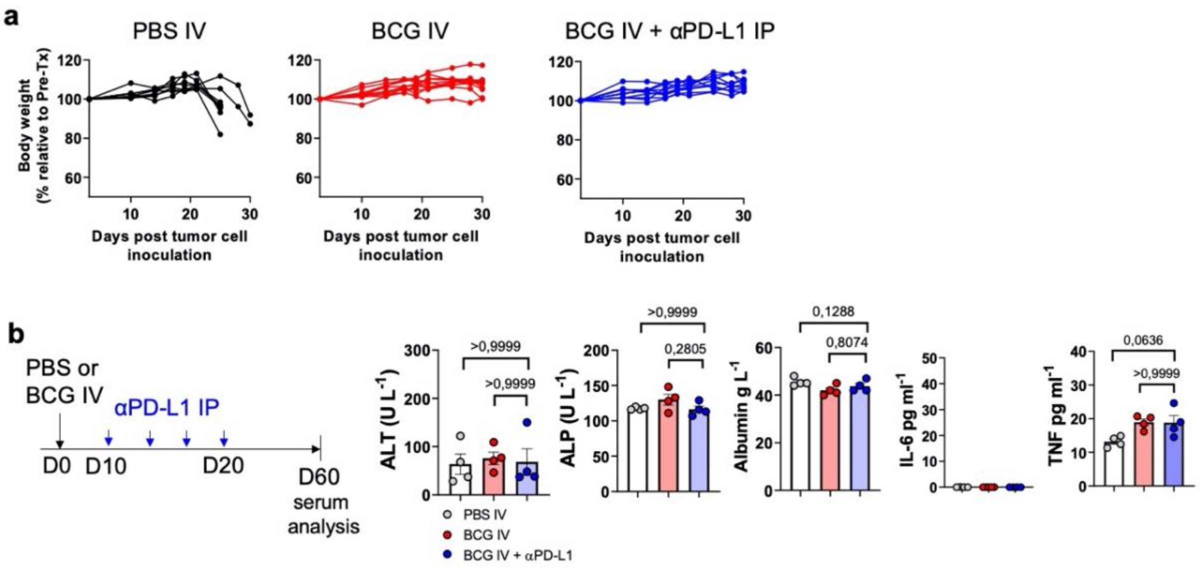

Supplementary Figure 12: Safety of IV BCG + PD-L1 blockade. a, Schematic diagram showing treatment strategy and follow-up of mice body weight in the indicated groups. Data comes from the survival experiment shown in Figure 9h. **b,** Schematic diagram showing treatment strategy and serum levels of ALT (alanine transaminase), ALP (alkaline phosphatase), albumin, IL-6 and TNF at the day 60 endpoint (n = 4 mice/group, from one experiment). *P* values were calculated using two-tailed unpaired Student's t-test at a 95 % CI (**b)** or one-way ANOVA with Bonferroni multiple-comparison test **(b)**. Data is depicted as mean +/- SEM. PBS: phosphate-buffered saline, PD-L1: programmed cell death ligand 1, IV: intravenous.

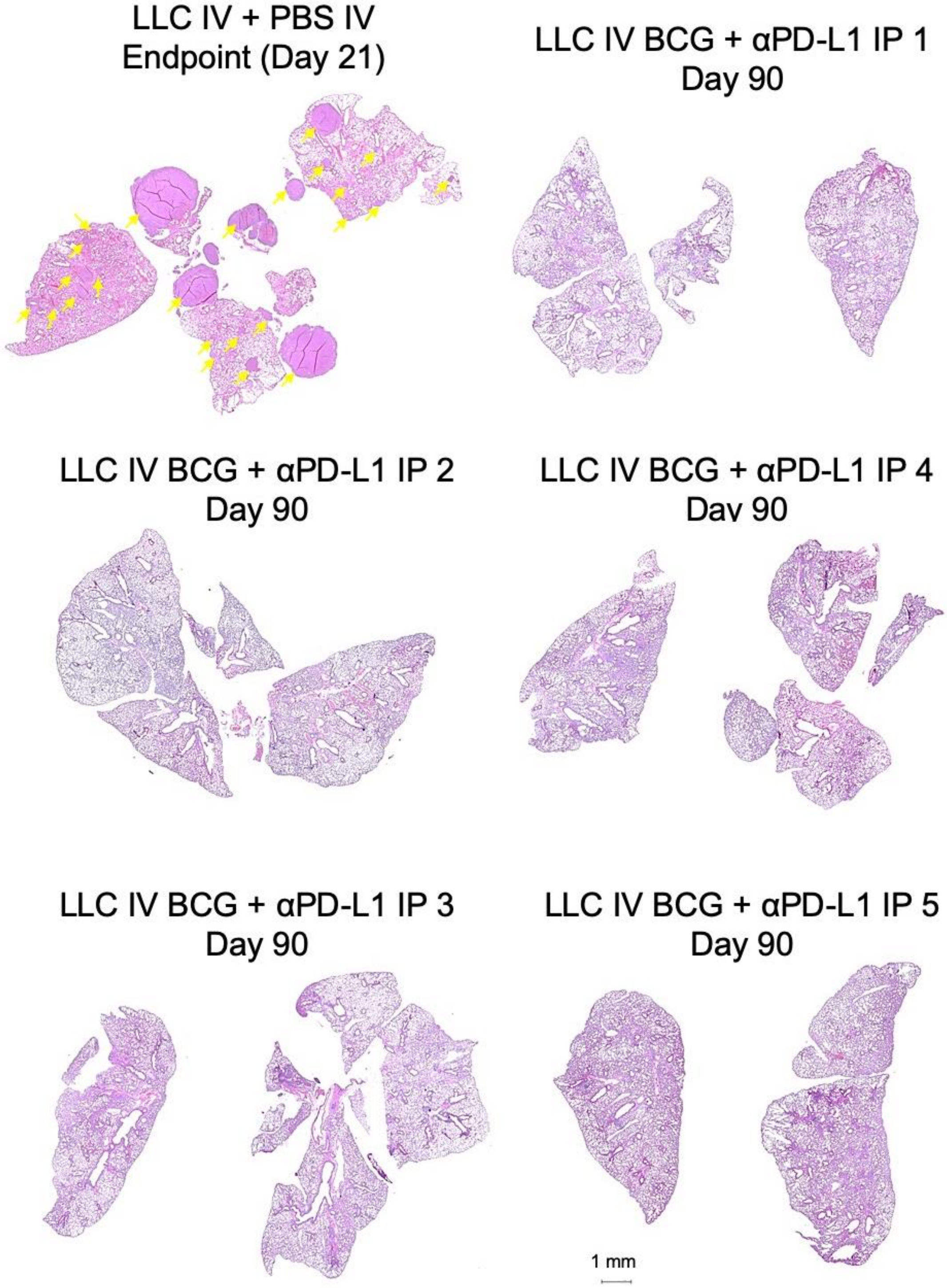

Supplementary Figure 13: H&E lung tissue sections of LLC survivors at day 90 post tumor cell inoculation. Shown are images of whole lung tissue after H&E staining. The first image shows the lungs of a mice bearing LLC lung tumors at day 21, at a humane endpoint stage for comparison, and yellow arrows indicate tumor nodules. The remaining 5 images show the lungs of mice inoculated with LLC tumor cells after IV BCG + antiPD-L1 treatment at day 90 post tumor cell inoculation, the predefined endpoint of experimental follow-up. PD-L1: programmed cell death ligand 1, IV: intravenous, IP: intraperitoneal.

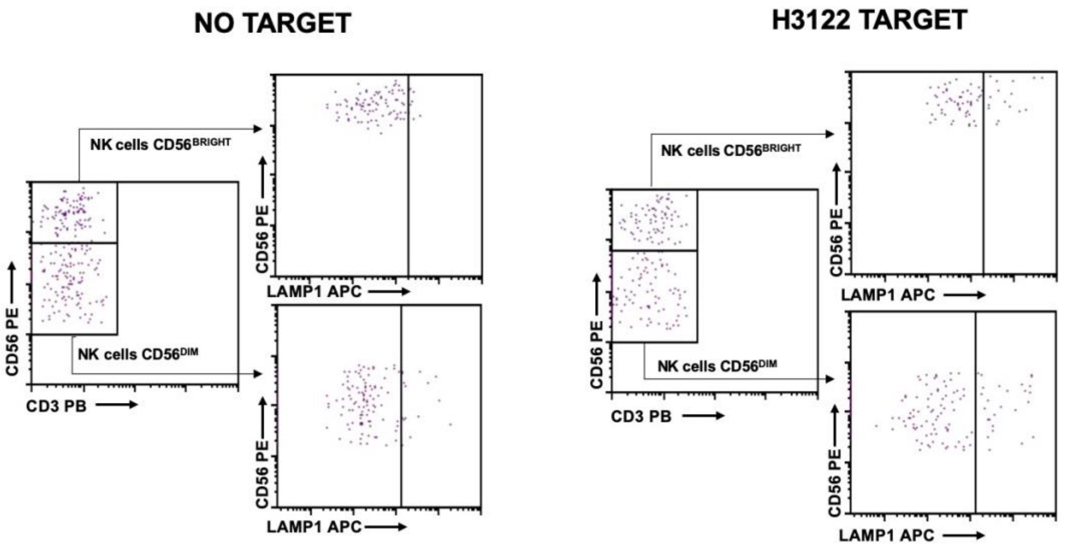

Supplementary Figure 14: Flow cytometry gating strategies for Lamp1 expression by human NK cells in cytotoxicity assays.

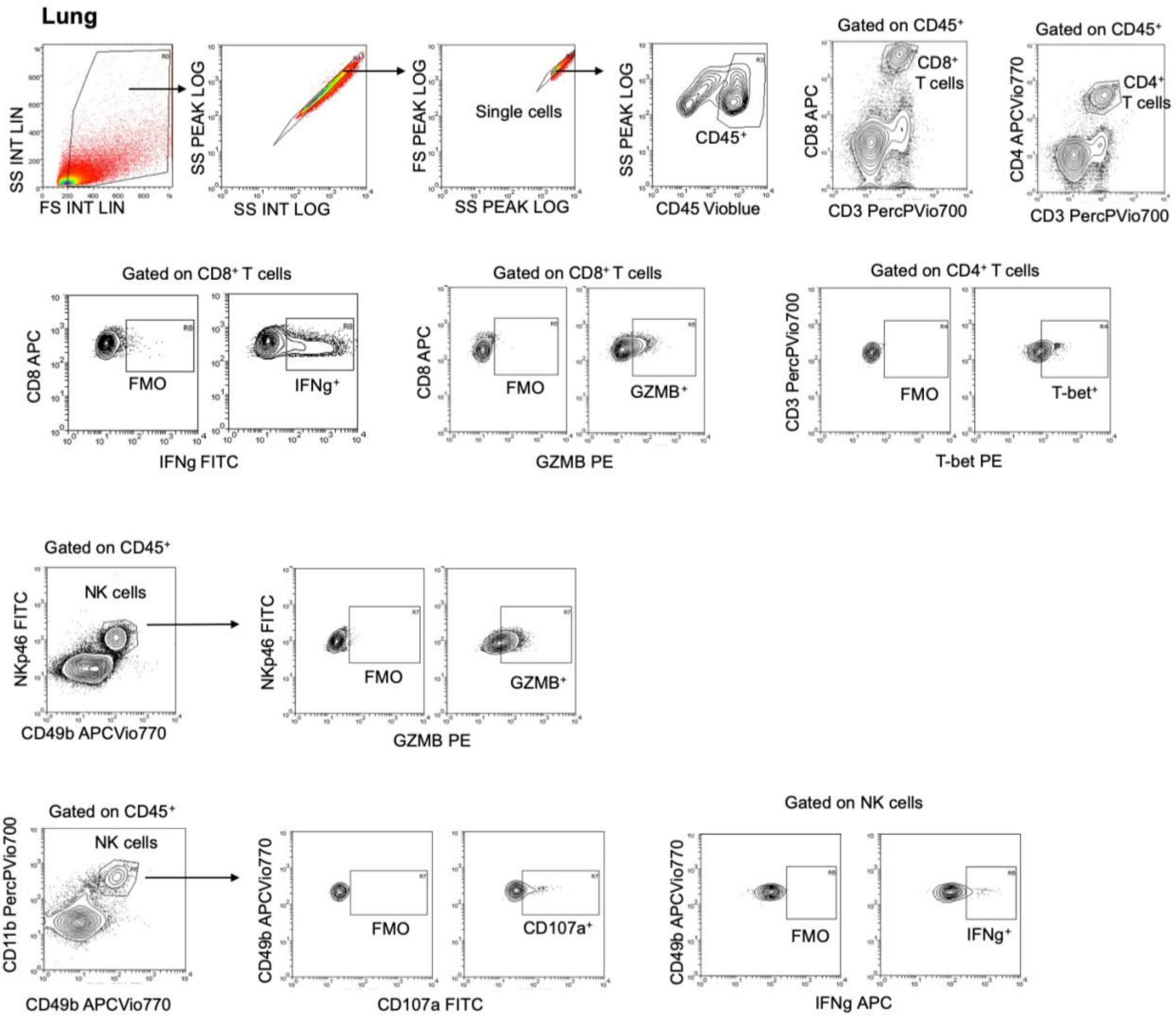

Supplementary Figure 15: Flow cytometry gating strategies (Gallios)

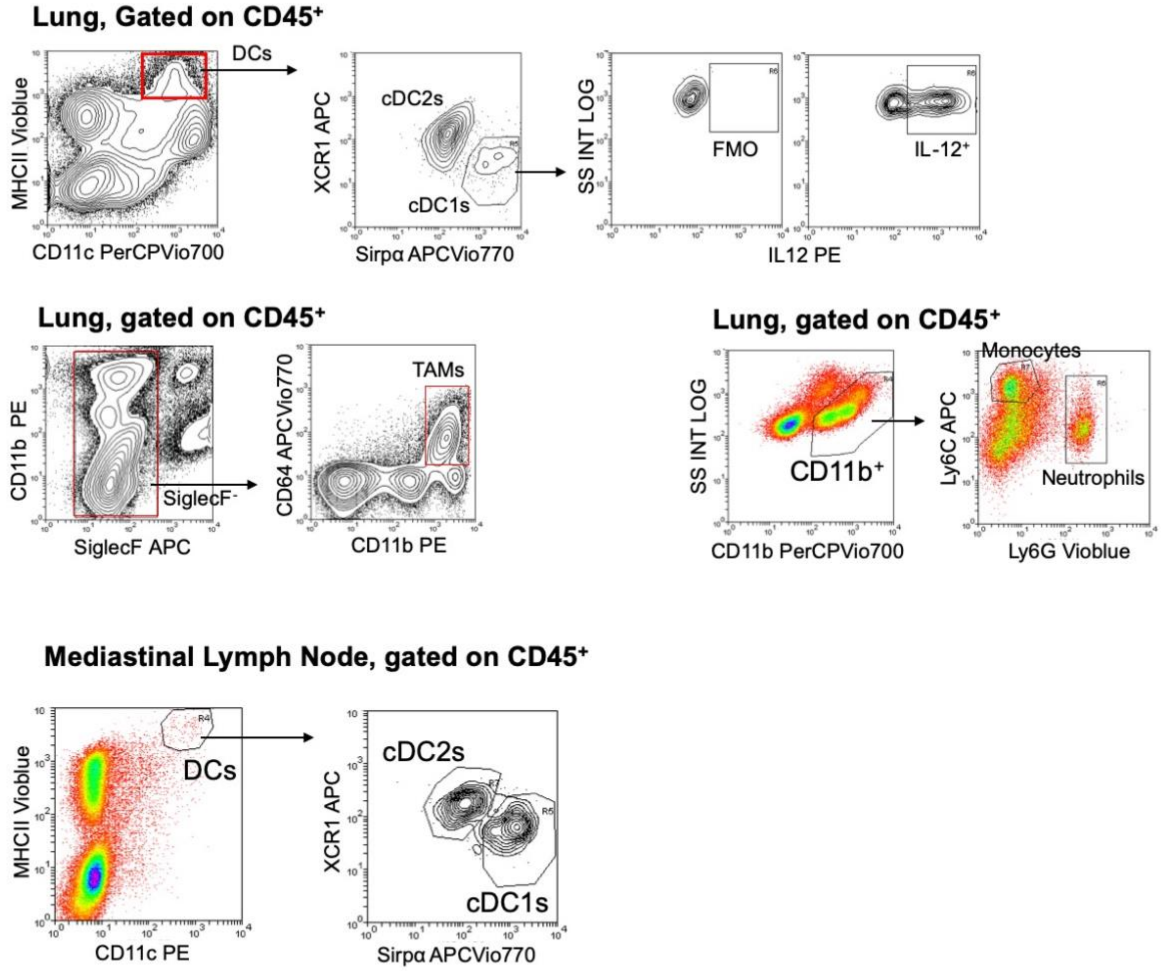

Supplementary Figure 16: Flow cytometry gating strategies (Gallios). DCs: Dendritic Cells.

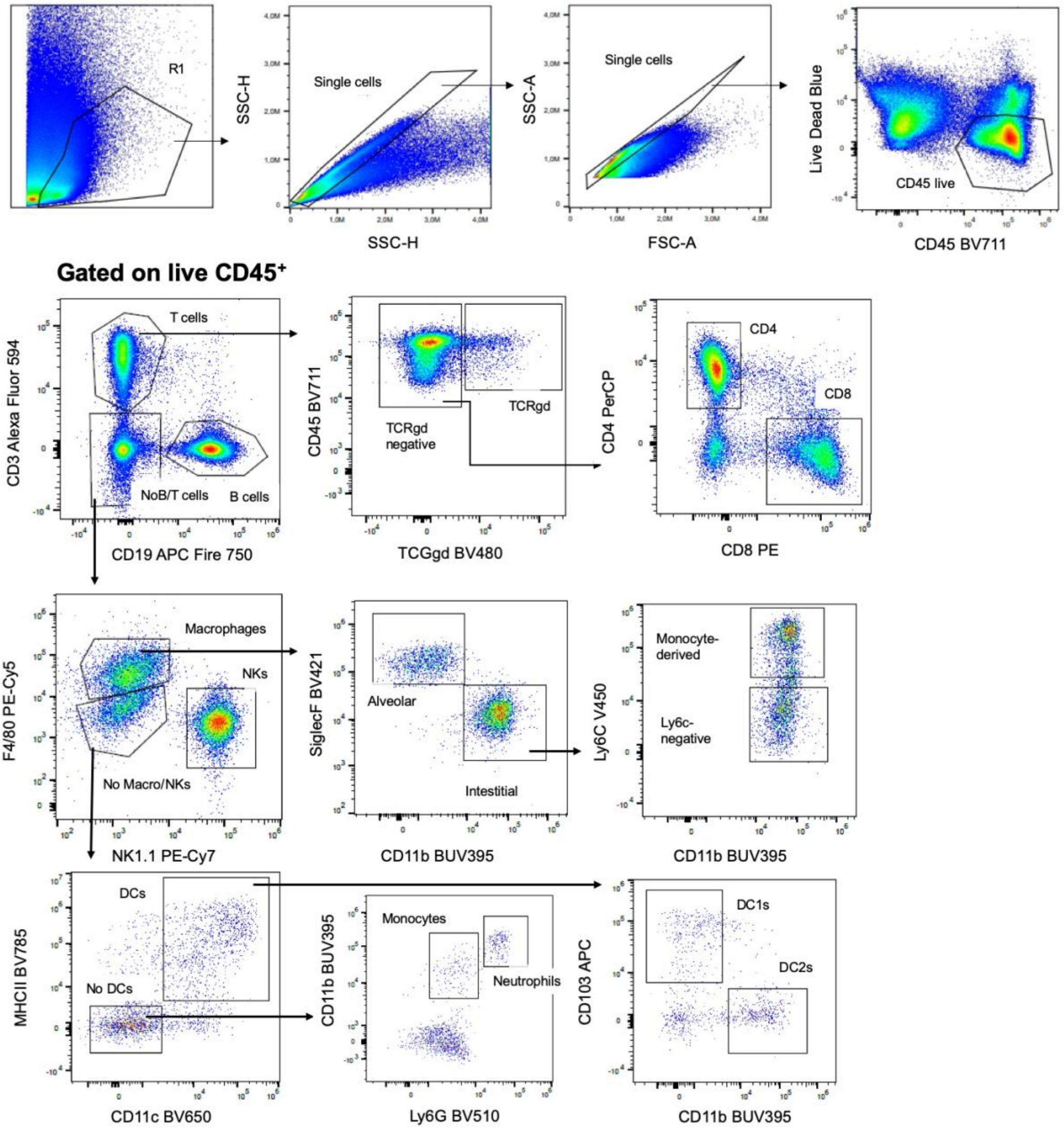

Supplementary Figure 17: Flow cytometry gating strategies (Spectral flow cytometry panel). TCRgd: TCR gamma delta; DCs: Dendritic Cells.

Supplementary Table 1. List of fluorochrome-conjugated antibodies used for conventional flow cytometry

ITEM	Fluorochrome	CLONE	PROVIDER	DILUTION
CD45	Vioblue	REA737	Miltenyi	1/100
CD45	PerCPVio700	REA737	Miltenyi	1/100
CD11b	PerCPVio700	REA592	Miltenyi	1/200
CD11c	PE	REA754	Miltenyi	1/200
CD11c	FITC	REA754	Miltenyi	1/200
F4/80	PE	REA126	Miltenyi	1/200
XCR1	APC	REA707	Miltenyi	1/200
MHCII	Vioblue	REA813	Miltenyi	1/100
H2Kb/Db	APC	REA932	Miltenyi	1/200
CD3	PerCPVio700	REA641	Miltenyi	1/200
CD4	FITC	REA604	Miltenyi	1/200
CD4	APCVio770	REA604	Miltenyi	1/200
CD8	PE	REA601	Miltenyi	1/200
CD8	APC	REA601	Miltenyi	1/200
CD86	PE	REA1190	Miltenyi	1/100
CD86	VioBright	REA1190	Miltenyi	1/100
CD40	VioBright	REA965	Miltenyi	1/100
CD172α	APCVio770	REA1201	Miltenyi	1/200
NKp46	PE	REA815	Miltenyi	1/200
CD64	APCVio770	REA286	Miltenyi	1/200
SiglecF	APC	REA798	Miltenyi	1/300
Ly6C	APC	REA796	Miltenyi	1/200
PD-L1	PE	MIH5	BD	1/100
Ly6G	Vioblue	REA526	Miltenyi	1/100
CD49b	APCVio770	DX5	Miltenyi	1/100
TCRg/d	APCVio770	GL3	BD	1/200
Granzyme B	PE	REA226	Miltenyi	1/50
T-bet	PE	REA102	Miltenyi	1/100
GATA3	APC	REA174	Miltenyi	1/100
CCL5	PE	2E9/CCL5	BD	1/100
IFN-γ	FITC, APC	REA638	Miltenyi	1/50
CD107a	FITC	1DB4	BD	2 µl/well
IL-12	PE	REA136	Miltenyi	1/10
IL-2	FITC	JES6-5H4	BD	1/100

Supplementary Table 2. List of fluorochrome-conjugated antibodies used for spectral flow cytometry

ITEM	Fluorochrome	CLONE	PROVIDER	DILUTION
LIVE/DEAD Fixable Blue			ThermoFisher	
BD Horizon Brilliant staining buffer			BD	
Purified anti-mouse CD16/CD32 (Fc Shield)		2,4G2	Tonbo	1/50
Ly6G	BV510	1A8	Biolegend	1/200
CD103	APC	2E7	Biolegend	1/200
Siglec-F	BV421	S17007L	Biolegend	1/200
CD4	PerCP	GK1,5	Biolegend	1/200
PD-1	BV605	J43	BD	1/200
CD44	BV570	IM7	Biolegend	1/200
TCRgd	BV480	GL3	BD	1/200
CD11b	BUV395	M1/70	BD	1/200
Ly6C	V450	AL-21	BD	1/200
F4/80	PECy5	BM8	Biolegend	1/200
CD3	AF594	500A2	Biolegend	1/200
CD11c	BUV650	N418	Biolegend	1/200
CD45	BV711	30-F11	BD	1/200
CD19	APCFire750	6D5	Biolegend	1/200
NK1.1	PECy7	PK136	Biolegend	1/200
CD8a	PE	53-6.7	Biolegend	1/200
I-A/I-E	BV785	M5/114.15.2	Biolegend	1/200
CD69	AF647	H1.2F3	Biolegend	1/200
CD62L	AF488	MEL-14	Biolegend	1/200
