# Supplementary material for: Intravenous administration of BCG in mice promotes natural killer and T cell-mediated antitumor immunity in the lung
Source: Nat Commun. 2023 Oct 4;14:6090. doi: 10.1038/s41467-023-41768-8 (PMC10551006; doi:10.1038/s41467-023-41768-8)
Supplement: Supplementary file 3 — Reporting Summary [file 41467_2023_41768_MOESM3_ESM.pdf]

nature portfolio

Corresponding author(s): NACHO AGUILO

Last updated by author(s): Aug 24, 2023

Reporting Summary

Nature Portfolio wishes to improve the reproducibility of the work that we publish. This form provides structure for consistency and transparency in reporting. For further information on Nature Portfolio policies, see our Editorial Policies and the Editorial Policy Checklist.

Statistics

For all statistical analyses, confirm that the following items are present in the figure legend, table legend, main text, or Methods section.

n/a	Confirmed	
☐	☒	The exact sample size (*n*) for each experimental group/condition, given as a discrete number and unit of measurement
☐	☒	A statement on whether measurements were taken from distinct samples or whether the same sample was measured repeatedly
☐	☒	The statistical test(s) used AND whether they are one- or two-sided<br>*Only common tests should be described solely by name; describe more complex techniques in the Methods section.*
☒	☐	A description of all covariates tested
☐	☒	A description of any assumptions or corrections, such as tests of normality and adjustment for multiple comparisons
☐	☒	A full description of the statistical parameters including central tendency (e.g. means) or other basic estimates (e.g. regression coefficient) AND variation (e.g. standard deviation) or associated estimates of uncertainty (e.g. confidence intervals)
☐	☒	For null hypothesis testing, the test statistic (e.g. *F*, *t*, *r*) with confidence intervals, effect sizes, degrees of freedom and *P* value noted<br>*Give P values as exact values whenever suitable.*
☒	☐	For Bayesian analysis, information on the choice of priors and Markov chain Monte Carlo settings
☒	☐	For hierarchical and complex designs, identification of the appropriate level for tests and full reporting of outcomes
☒	☐	Estimates of effect sizes (e.g. Cohen's *d*, Pearson's *r*), indicating how they were calculated

Our web collection on statistics for biologists contains articles on many of the points above.

Software and code

Policy information about availability of computer code

Data collection	For mouse experiments, flow cytometry data was collected using a Beckman Coulter Gallios flow cytometer. For spectral flow cytometry, samples were acquired in an Aurora Cytek 5L spectral flow cytometer with the SpectroFlo software. For experiments with human PBMCs, data was collected using a CytoFlex (Beckman Coulter) flow cytometer. For ELISA and luminiscence measurements, a Multi-Mode Microplate Reader (Synergy™ HT, BioTek) was used. Cell sorting was performed using a SH800S Cell Sorter (Sony).
Data analysis	Conventional Flow cytometry data from mouse experiments was analyzed with Weasel v3.0.2. Flow cytometry data from human experiments was analyzed using Kaluza software. For spectral flow cytometry, analysis of cellular populations was performed with FlowJo (TreeStar) v10.7.2 software, Visualization and statistical analysis was performed using GraphPad Prism 8 software.

For manuscripts utilizing custom algorithms or software that are central to the research but not yet described in published literature, software must be made available to editors and reviewers. We strongly encourage code deposition in a community repository (e.g. GitHub). See the Nature Portfolio guidelines for submitting code & software for further information.

Data

Policy information about availability of data

All manuscripts must include a data availability statement. This statement should provide the following information, where applicable:

- Accession codes, unique identifiers, or web links for publicly available datasets
- A description of any restrictions on data availability
- For clinical datasets or third party data, please ensure that the statement adheres to our policy

Raw data is provided as an Excel file in the Source Data section. Materials are available upon request to the authors.

Human research participants

Policy information about studies involving human research participants and Sex and Gender in Research.

Reporting on sex and gender	No gender information from healthy donors who provided PBMCs was provided by Transfusion Centre
Population characteristics	No information from healthy donors who provided PBMCs was provided by Transfusion Centre, with regard to population characteristics
Recruitment	PBMCs from buffy coats of healthy donors were obtained from the Regional Transfusion Centre (Madrid)
Ethics oversight	PBMCs from buffy coats of healthy donors were obtained from the Regional Transfusion Centre (Madrid) with ethical permission and experimental protocols approved by the institutional committees: Regional Transfusion Centre (PO-DIS-09) and assessed by the bioethics committee of CSIC. Informed consent was obtained at the Transfusion Centre from all participants. All methods were carried out in accordance with biosafety guidelines and regulations authorized by CNB-CSIC.

Note that full information on the approval of the study protocol must also be provided in the manuscript.

Field-specific reporting

Please select the one below that is the best fit for your research. If you are not sure, read the appropriate sections before making your selection.

☒ Life sciences ☐ Behavioural & social sciences ☐ Ecological, evolutionary & environmental sciences

For a reference copy of the document with all sections, see nature.com/documents/nr-reporting-summary-flat.pdf

Life sciences study design

All studies must disclose on these points even when the disclosure is negative.

Sample size	Statistical methods were not used to predetermine sample size. Sample size followed common standards in the field.<br><br>The number of animals used for each experiments was estimated based on previous and pilot studies. Number of animals for each experimental group are described in Figure legends.<br><br>For in vitro experiments, sample size was determined according to our previous experience, with experimental groups enough numerous to apply the corresponding statistical tests.
Data exclusions	Outliers were not excluded for statistical analysis.
Replication	Data supporting the main conclusions of the study are displayed as pooled data from at least two independent experiments, with at least 4 biological replicates per experimental group and experiment. Experiments performed once were done with at least 6 biological replicates per group. All attemps of replication were successful
Randomization	Tumors were induced at day 0 and mice were randomized into different experimental groups at day 7, prior to treatment application. For in vitro experiments, samples were randomly allocated into the different experimental groups.
Blinding	Animal monitoring and data analysis were not blinded, since generally the whole experiment and analysis were performed by the same person

Reporting for specific materials, systems and methods

We require information from authors about some types of materials, experimental systems and methods used in many studies. Here, indicate whether each material, system or method listed is relevant to your study. If you are not sure if a list item applies to your research, read the appropriate section before selecting a response.

Materials & experimental systems

n/a	Involved in the study
☐	☒ Antibodies
☐	☒ Eukaryotic cell lines
☒	☐ Palaeontology and archaeology
☐	☒ Animals and other organisms
☒	☐ Clinical data
☒	☐ Dual use research of concern

Methods

n/a	Involved in the study
☒	☐ ChIP-seq
☐	☒ Flow cytometry
☒	☐ MRI-based neuroimaging

Antibodies

Antibodies used

For mouse experiments, antibodies for conventional and spectral flow cytometry were:

Target/Fluorochrome/CLONE/PROVIDER/DILUTION

CD45/ Vioblue/ REA737/ Miltenyi/ 1/100
CD45/ PerCPVio700/ REA737/ Miltenyi/ 1/100
CD11b/ PerCPVio700/ REA592/ Miltenyi/ 1/200
CD11c/ PE/ REA754/ Miltenyi/ 1/200
CD11c/ FITC/ REA754/ Miltenyi/ 1/200
F4/80/ PE/ REA126/ Miltenyi/ 1/200
XCR1/ APC/ REA707/ Miltenyi/ 1/200
MHCII/ Vioblue/ REA813/ Miltenyi/ 1/100
H2Kb/Db/ APC/ REA932/ Miltenyi/ 1/200
CD3/ PerCPVio700/ REA641/ Miltenyi/ 1/200
CD4/ FITC/ REA604/ Miltenyi/ 1/200
CD4/ APCVio770/ REA604/ Miltenyi/ 1/200
CD8/ PE/ REA601/ Miltenyi / 1/200
CD8/ APC/ REA601/ Miltenyi / 1/200
CD86/ PE/ REA1190 / Miltenyi/ 1/100
CD86/ VioBright/ REA1190/ Miltenyi / 1/100
CD40/ VioBright/ REA965/ Miltenyi / 1/100
CD172α/ APCVio770/ REA1201/ Miltenyi / 1/200
NKp46/ PE / REA815/ Miltenyi/ 1/200
CD64/ APCVio770/ REA286/ Miltenyi/ 1/200
SiglecF/ APC/ REA798/ Miltenyi/ 1/300
Ly6C/ APC/ REA796/ Miltenyi /1/200
PD-L1 / PE/ MIH5/ BD/ 1/100
Ly6G/ Vioblue / REA526/ Miltenyi/ 1/100
CD49b/ APCVio770/ DX5/ Miltenyi/ 1/100
TCRg/d/ APCVio770/ GL3/ BD/ 1/200
Granzyme B/ PE/ REA226/ Miltenyi/ 1/50
T-bet / PE/ REA102/ Miltenyi/ 1/100
GATA3/ APC/ REA174/ Miltenyi/ 1/100
CCL5/ PE/ 2E9_CCL5/ BD/ 1/100
IFN-γ /FITC/ REA638/ Miltenyi/ 1/50
IFN-γ /APC/ REA638/ Miltenyi/ 1/50
CD107a/ FITC/ 1DB4/ BD/ 2 μl/well
IL-12/ PE/ REA136/ Miltenyi/ 1/10
IL-2/ FITC/ JES6-5H4/ BD/ 1/100
Ly6G/ BV510/ 1A8/ Biolegend/ 1/200
CD103/ APC/ 2E7/ Biolegend/ 1/200
Siglec-F/ BV421/ S17007L/ Biolegend/ 1/200
CD4/ PerCP/ GK1,5/ Biolegend/ 1/200
PD-1/ BV605/ J43/ BD/ 1/200
CD44/ BV570/ IM7/ Biolegend/ 1/200
TCRgd/ BV480/ GL3/ BD/ 1/200
CD11b/ BUV395/ M1_70/ BD/ 1/200
Ly6C/ V450/ AL-21/ BD/ 1/200
F4/80/ PECy5/ BM8/ Biolegend/ 1/200
CD3/ AF594/ 500A2/ Biolegend/ 1/200
CD11c/ BUV650/ N418/ Biolegend/ 1/200
CD45/ BV711/ 30-F11/ BD/ 1/200
CD19/ APCFire750/ 6D5/ Biolegend/ 1/200
NK1.1/ PECy7/ PK136/ Biolegend/ 1/200
CD8a/ PE/ 53-6.7/ Biolegend/ 1/200
I-A/I-E/ BV785/ M5_114.15.2/ Biolegend/ 1/200

CD69/ AF647/ H1.2F3/ Biolegend/ 1/200
CD62L/ AF488/ MEL-14/ Biolegend/ 1/200

For human experiments, antibodies for flow cytometry were: CD3-PB (clone UCHT1), CD16-PE-Cy7 (clone 3G8), CD56-PE (clone MEM.188 and Lamp-1-APC (clone H4-A3) from Biolegend. Human sample assays were done adding 0.1ug of the corresponding antibody per 0.5 million cells, except Lamp-1 assays, in which 0.2ug were added.

For in vivo depletion experiments, anti-CD4 (clone GK1.5), anti-CD8 (clone 2.43) and antiNK1.1 (clone PK136) were used, from BioXCell.

For treatments, anti-PD-L1 (clone 10F.9G2) was used, from BioXCell.

Validation	All antibodies used in the study are commercially avalaible and validated by the manufacturer. Validation data are available at the manufacturer´s website. All antibody clones described here have been used used across many studies and laboratories.

Eukaryotic cell lines

Policy information about cell lines and Sex and Gender in Research

Cell line source(s)	B16-F10 and B16-F10-gp33 cells were given by Dr. Julián Pardo (University of Zaragoza). LLC cells were given by Dr. David Sancho (Centro Nacional de Investigaciones Cardiovasculares, Madrid). LLC-OVA tumor cells were kindly provided by Dmitry Gabrilovich. TC-1 cells were given by Dr. T.C. Wu (Johns Hopkins Medicine). B16-F10-ZsGreenLuc and LLC-ZsGreenLuc cells were made in the laboratory by transfection with a lentivirus encoding ZsGreen and firefly luciferase Luc2P (pHIV-Luc2-ZsGreen, from Addgene) and sorted based on high ZsGreen expression. For the generation of LLC-B2m-/- and B16-F10-B2m-/- cell lines, parental cells were transfected with CRISPR-Cas9 plasmids targeting the β2-microglobulin gene (purchased from SantaCruz Biotechnology) and cells were selected with puromycin and then sorted based on lack of MHC-I expression after staining with an antibody directed to H2Kb/Db (Miltenyi). Tumor cells were cultured with complete DMEM, containing 10% inactivated Fetal Bovine Serum (FBS, Gibco), Glutamax (Gibco) and penicillin/streptomycin (Gibco) and were always used with less than 8 passages from thawing. The B16-F10-gp33 and LLC-OVA clones were maintained in complete DMEM containing 500 µg ml-1 of G418 (Gibco).<br>The human lung cancer cell lines H1322 (bronchi-alveolar carcinoma) and H2188 (lung adenocarcinoma) were obtained from Dr. A. Romero (Puerta de Hierro Hospital, Madrid) and authenticated by satellite genotyping at the genomics service of the Instituto de Investigaciones Biomédicas (IIB-CSIC).
Authentication	The human lung cancer cell lines H1322 and H2188 were authenticated by satellite genotyping . Mouse cell lines were not authenticated.
Mycoplasma contamination	Cells were routinely tested for mycoplasma and no mycoplasma contamination was detected.
Commonly misidentified lines<br>(See ICLAC register)	No misidentified lines were used in the study

Animals and other research organisms

Policy information about studies involving animals; ARRIVE guidelines recommended for reporting animal research, and Sex and Gender in Research

Laboratory animals	Male and female mice between the ages of 8 and 12 weeks were used. C57BL/6JR mice were purchased to Janvier Biolabs. Mouse strains deficient for interferon gamma (IFNγ-/-, strain #002287) and Rag1 (Rag1-/-, strain #002216) bred on C57BL/6JR background were purchased from Jackson Laboratories. The mouse strains deficient for Perforin (Perf-/-)69 and Batf3 (Batf3-/-)70 on C57BL/6JR background were bred in the facilities of the Centro de Investigaciones Biomédicas de Aragón (CIBA). Mouse experimentation and breeding were done in a SPF-facility at 20-24 ºC, 50-70 % humidity and a light-dark cycle of 12 hours.
Wild animals	No wild animals were used in the study
Reporting on sex	For experiments involving B16F10 tumor models, they were cojnducted in female mice as strong differences between male and female were observed previously in pilot studies, finding that male mice generated a lower number of metastasis upon intravenous administration of B16-F10 cells. In the case of LLC lung tumor models, male and female mice were used since no differences were found between them.
Field-collected samples	The study did not involve samples collected from the field
Ethics oversight	Experimental work was conducted in agreement with European and national directives for protection of experimental animals, and experimental procedures were approved by the Ethics Committee for Animal Experiments of University of Zaragoza (PI46/18, PI33/15 and PI50/14).

Note that full information on the approval of the study protocol must also be provided in the manuscript.

Flow Cytometry

Plots

Confirm that:

☒ The axis labels state the marker and fluorochrome used (e.g. CD4-FITC).

☒ The axis scales are clearly visible. Include numbers along axes only for bottom left plot of group (a 'group' is an analysis of identical markers).

☒ All plots are contour plots with outliers or pseudocolor plots.

☒ A numerical value for number of cells or percentage (with statistics) is provided.

Methodology

Sample preparation	Lungs were aseptically removed and homogenized in DMEM containing deoxyribonuclease I (DNase I, 40 U ml-1; AppliChem) and collagenase D (2 mg ml-1; Roche) using a GentleMacs dissociator (Miltenyi Biotec) according to manufacturer's instructions. Lungs were incubated at 37°C for 30 min and further homogenized with the GentleMacs dissociator. The homogenates were filtered through a 70 µm cell strainer (MACS SmartStainers, Miltenyi Biotec). Erythrocytes were lysed with RBC Lysing Buffer for 1 min and single cells were resuspended in PBS with 2% FBS and 1 mM EDTA and stained for surface and intracellular markers. Spleens and lymph nodes were mashed with the back of a syringe in RPMI with 2 mg ml-1 Collagenase D and 40 U ml-1 DNase I, incubated for 20 min at 37ºC and strained through a 70 µM cell strainer before lysing erythrocytes with RBC Lysing Buffer for 1 min.
Instrument	For mouse experiments, flow cytometry data was collected using a Beckman Coulter Gallios flow cytometer. For spectral flow cytometry, samples were acquired in an Aurora Cytek 5L spectral flow cytometer with the SpectroFlo software. For experiments with human PBMCs, data was collected using a CytoFlex (Beckman Coulter) flow cytometer. For ELISA and luminiscence measurements, a Multi-Mode Microplate Reader (Synergy™ HT, BioTek) was used. Cell sorting was performed using a SH800S Cell Sorter (Sony).
Software	Conventional Flow cytometry data from mouse experiments was analyzed with Weasel v3.0.2. Flow cytometry data from human experiments was analyzed using Kaluza software. For spectral flow cytometry, analysis of cellular populations was performed with FlowJo (TreeStar) v10.7.2 software.
Cell population abundance	n/a
Gating strategy	Gating strategies are shown in Extended data Figures 15, 16, 17 and 18

☒ Tick this box to confirm that a figure exemplifying the gating strategy is provided in the Supplementary Information.
